# Clinical Efficacy and Safety of Vancomycin Continuous Infusion in Patients Treated at Home in an Outpatient Parenteral Antimicrobial Therapy Program

**DOI:** 10.3390/antibiotics11050702

**Published:** 2022-05-23

**Authors:** Lore Thijs, Charlotte Quintens, Lotte Vander Elst, Paul De Munter, Melissa Depypere, Willem-Jan Metsemakers, Georges Vles, Astrid Liesenborghs, Jens Neefs, Willy E. Peetermans, Isabel Spriet

**Affiliations:** 1 Pharmacy Department, University Hospitals Leuven, 3000 Leuven, Belgium; lore.1.thijs@uzleuven.be (L.T.); lotte.vanderelst@uzleuven.be (L.V.E.); astrid.liesenborghs@uzleuven.be (A.L.); jens.neefs@uzleuven.be (J.N.); isabel.spriet@uzleuven.be (I.S.); 2 Department of Pharmaceutical and Pharmacological Sciences, KU Leuven, 3000 Leuven, Belgium; 3 Department of Microbiology, Immunology and Transplantation, KU Leuven, 3000 Leuven, Belgium; paul.demunter@uzleuven.be (P.D.M.); willy.peetermans@uzleuven.be (W.E.P.); 4 Department of General Internal Medicine, University Hospitals Leuven, 3000 Leuven, Belgium; 5 Clinical Department of Laboratory Medicine, University Hospitals Leuven, 3000 Leuven, Belgium; melissa.depypere@uzleuven.be; 6 Department of Trauma Surgery, University Hospitals Leuven, 3000 Leuven, Belgium; willem-jan.metsemakers@uzleuven.be; 7 Department of Development and Regeneration, KU Leuven, 3000 Leuven, Belgium; 8 Department of Orthopaedics, University Hospitals Leuven, 3000 Leuven, Belgium; georges.vles@uzleuven.be

**Keywords:** OPAT at home, vancomycin, continuous infusion, elastomeric pump, therapeutic drug monitoring, adverse event monitoring, satisfaction survey

## Abstract

Vancomycin is commonly used in outpatient parenteral antimicrobial therapy (OPAT) of Gram-positive infections. Therapeutic drug monitoring and adverse event monitoring pose a challenge. Outcome data of vancomycin in OPAT (vOPAT) are limited. The study aim was to report the safety and efficacy of a structured vOPAT program implemented in the University Hospitals Leuven. The program provides continuous elastomeric infusion of vancomycin at home with biweekly follow-up at the outpatient clinic. Demographics, clinical, biochemical and treatment parameters, target attainment parameters and clinical outcomes were recorded. An e-survey was conducted to assess patient satisfaction. Thirty-five vOPAT episodes in 32 patients were included. During 206 follow-up consultations, 203 plasma concentration measurements were registered with a median vancomycin plasma concentration of 22.5 mg/L (range 6.6–32.0). The majority of concentrations (68.5%) were within the therapeutic range (20.0–25.0 mg/L). Adverse event rates, including drug- (5.7%) and catheter-related (5.7%) events, were low. For 32 vOPAT episodes, a clinical cure rate of 100% was observed. All patients who completed the e-survey were satisfied with their vOPAT course. These findings show that a structured vOPAT program with rigorous follow-up provides safe and effective ambulatory treatment of patients with vancomycin in continuous infusion.

## 1. Introduction

Outpatient parenteral antimicrobial therapy (OPAT) can be a feasible option for patients who need long-term treatment with intravenous antimicrobials, but are deemed clinically well enough to allow continuation of treatment in an ambulatory setting, e.g., at the patient’s home [1]. OPAT offers several advantages for both the patient and health care systems, such as improved quality of life, earlier functional recovery, decreased length of hospital stay and reduced risk of nosocomial infections [2,3,4]. Moreover, OPAT has proven its safety and clinical efficacy in multiple studies, and patients receiving OPAT services report improved satisfaction [1,2,4,5].

Vancomycin is a glycopeptide antibiotic that, when intended for systemic treatment, should be administered intravenously due to its poor gastrointestinal absorption. It is used for the treatment of infections with Gram-positive bacteria, such as methicillin-resistant *Staphylococcus aureus*, coagulase-negative staphylococci and ampicillin-resistant *Enterococcus* species. Clinical indications include periprosthetic joint infections, fracture-related infections, native osteomyelitis, complicated skin and soft tissue infections and endocarditis [6,7,8,9]. These indications generally require a long course of antibiotic treatment, with recommended therapy durations ranging from several weeks to several months [10,11,12]. Once the patient is clinically well, therapy with an oral antimicrobial agent should be the preferred choice for treatment continuation. Unfortunately, structural or functional problems hampering the absorption of antimicrobial agents, low bio-availability of the oral antimicrobial alternative itself or, in most cases, resistance of micro-organisms to oral antimicrobial alternatives can render this option invalid [5,13,14]. In these conditions, vancomycin in OPAT (vOPAT) may be a valid alternative. However, the use of vOPAT poses new challenges, such as the need for therapeutic drug monitoring (TDM) and close follow-up of adverse events [4].

Vancomycin has a narrow therapeutic index. To ensure efficacy and avoid toxicity, TDM is needed, targeting a ratio of area under the curve to minimum inhibitory concentration (AUC/MIC) between 400 and 600. This target is often monitored by measuring either trough or steady-state concentrations [6,15,16]. The optimal frequency of monitoring is undefined and should be based on clinical judgement, with guidelines suggesting at least once-weekly monitoring of plasma concentrations for hemodynamically stable patients [15,16,17]. TDM in the outpatient setting can be challenging due to a variety of reasons such as patient immobility, poor access to laboratory facilities, poor transmural communication and geographic isolation [3]. Next to TDM, clinical and biochemical surveillance of potential adverse events is essential when patients are treated outside the closely monitored environment of the hospital [2,4,5,18,19]. Vancomycin-specific adverse drug events (ADEs) include local phlebitis, nephrotoxicity and eosinophilia. Line-related adverse events (LRAEs) can also occur as OPAT-related adverse events [8,15]. In addition, vancomycin is reported to be an independent predictor for developing ADEs in OPAT [4,18].

Despite these challenges, guidelines specifically addressing the organization of vancomycin in OPAT programs are limited. Only a few approaches have been described in the literature, with examples including a nurse-coordinated home-based follow-up or implementation of a clinical pharmacy service for monitoring therapy [3,20]. Furthermore, there is a paucity of studies reporting on the outcome and safety of vOPAT. Therefore, the purpose of this study was to describe and evaluate the efficacy and safety of vOPAT within a structured OPAT program.

## 2. Materials and Methods

### 2.1. Design, Setting and Study Participants

This mono-center, prospective observational study was carried out in a 1950-bed tertiary care center in Belgium. All patients who were treated with vancomycin in the OPAT program and whose follow-up was completely coordinated by the OPAT team between January 2017 and August 2021 were included in the study. Approval for this study was granted by the institutional national research committee (Ethics Committee Research University Hospitals Leuven, Belgium; S60847).

### 2.2. Organization of the vOPAT Care Path within a Structured Program

The OPAT program of the University Hospitals Leuven was described previously [21]. In brief, the OPAT service is a home-based program, where home care nurses provide administration of antimicrobials at the patient’s home. The OPAT program is based on a multidisciplinary collaboration between the treating physician, the infectious disease specialist, the microbiologist, clinical pharmacists and (home care) nurses, and is coordinated by a clinical pharmacist who is also referred to as the OPAT coordinator.

**Figure 1:**
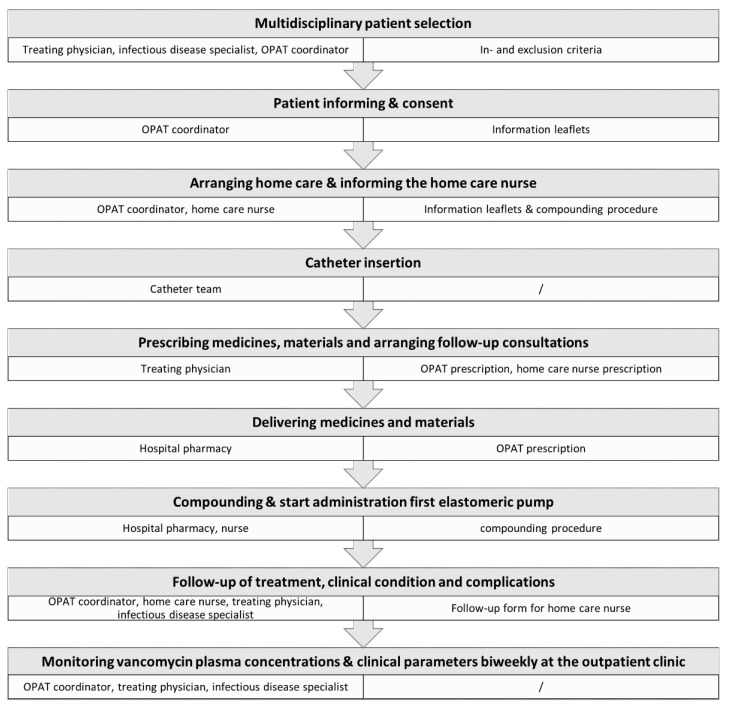
Flow of the structured vOPAT care path of the University Hospitals Leuven.

The structured vOPAT care path (Figure 1) is initiated by the treating physician identifying a potential vOPAT candidate based on predefined inclusion and exclusion criteria (Table 1). Consequently, discharge with vOPAT should be approved by the infectious disease specialist. Next, the OPAT coordinator verifies the patient-specific criteria (Table 1) and informs the patient. Written information is additionally provided in the form of information leaflets.

Continuous infusion of vancomycin over 24 h with elastomeric pumps (Baxter, Infusor^®^, Deerfield, IL, USA) is the exclusive mode of administration in this OPAT program. Therefore, patients treated with intermittent vancomycin infusion during hospitalization are switched to continuous infusion prior to discharge. Given the high likelihood of phlebitis and vascular irritation associated with peripheral vancomycin administration, a central venous line, such as a peripherally inserted central catheter (PICC) or a venous port, is preferred. Discharge is only allowed if the last two vancomycin plasma concentrations under continuous infusion are in the therapeutic range (20–25 mg/L). The treating physician is responsible for prescribing the therapy and planning of the follow-up consultations. The first elastomeric pump for vOPAT is compounded by the hospital pharmacy.

At home, the home care nurse visits the patient once a day for compounding and administration of the elastomeric pump. Information leaflets regarding vancomycin dosing and potential adverse events, catheter care, compounding and administration of elastomeric pumps are provided to the home care nurse. The home care nurse is also requested to fill in a follow-up form at every visit, documenting parameters such as weight of the elastomeric pump before and after infusion, ADEs and body temperature. The completed form is reviewed by the OPAT coordinator on a weekly basis.

After discharge, patients have follow-up consultations at the outpatient clinic every Tuesday and Friday for a mid-term evaluation. During each visit, vancomycin plasma concentrations, as well as C-reactive protein concentrations and renal function are monitored. Based on these measurements, dose adjustments are prescribed using a dose adjustment protocol provided by the OPAT team (Table 2). Dose adjustments are then communicated to the home care nurse and will be applied during the next administration at home. In case of an urgent dose adjustment (plasma concentrations > 27.5 mg/L or <17.5 mg/L), a new elastomeric pump is immediately compounded by the hospital pharmacy.

At discharge and after every follow-up consultation, the hospital pharmacy provides the patient with the necessary materials and vials for compounding the elastomeric pumps until the next follow-up appointment. An end-of-therapy consultation is foreseen by the treating physician at the hospital to ensure removal of the central venous line and for assessing clinical outcome.

### 2.3. Data Collection and Analysis

All vOPAT episodes were prospectively registered in a database. Some patients received multiple vOPAT episodes, which were reviewed independently. Demographic data such as age, gender, weight and renal function at the start of vancomycin therapy were registered. Renal function was expressed as the estimated Glomerular Filtration Rate (eGFR) calculated according to the CKD-EPI formula. The following clinical data were extracted: type of infection, causative micro-organism, medical discipline, length of hospital stay, (reasons for) unplanned readmissions and clinical outcome. Clinical outcome of vOPAT was rated as either “cure” or “failure”. Patients were assessed as clinically cured at the end of therapy with vOPAT in the case of absence of fever or local signs of infection and if there were no unplanned hospital readmissions for the same clinical problem, as well as no registration of the same infection up to one month after completion of vOPAT. Clinical failure was determined as relapse of infection during or within one month after completion of vOPAT. Patients temporarily readmitted, whether unplanned or not, due to vOPAT- or non-vOPAT-related problems and who nonetheless finished their vOPAT episode were still assessed as either clinically cured or failed according to the abovementioned definition. Patients were registered as non-assessable in case of early discontinuation of vOPAT caused by vOPAT-related adverse events or unplanned readmission for reasons other than the infection under treatment. The following treatment details were documented: duration of vOPAT, type of venous access device, vancomycin plasma concentrations, dosing regimen and dose adjustments, adherence to dose adjustment protocol, time to first therapeutic plasma concentration since start of therapy and when switching from intermittent to continuous infusion. Vancomycin plasma concentrations ranging from 20 to 25 mg/L were considered therapeutic for continuous infusion. Plasma concentrations were measured by a validated immunoassay using a Cobas analyzer c502 (Roche Diagnostics^®^, Basel, Switzerland). Additionally, vOPAT-related adverse events, including ADEs and LRAEs, were registered. ADEs were defined as any untoward medical occurrence in a patient associated, whether causally or not, with administration of vancomycin.

In order to assess patients’ satisfaction and overall experience with this vOPAT program, a brief 5-question e-survey was conducted via Google Forms. The content of the survey was set up by two clinical pharmacists (C.Q., L.V.E.) and validated by a senior clinical pharmacist (I.S.). The survey was sent to patients who completed an vOPAT episode between January 2018 and October 2021 and whose e-mail address was registered in the electronic health record.

## 3. Results

### 3.1. Demographics

Thirty-five vOPAT episodes in a total of 32 patients were included. The median age of patients was 61 years (range 11–75), and 65.7% of patients were male. There were two pediatric patients among the study population. Median renal function at the start of the vancomycin therapy was 90.0 mL/min/1.73 m^2^ (range 45.0–188.0). Augmented renal clearance, defined as an eGFR of more than 96.5 mL/min/1.73 m^2^ [22], was present in 11 patients (median eGFR 102.0 mL/min/1.73 m^2^; range 97.0–188.0). The median weight of the study participants was 82.0 kg (range 39.0–140.0). Demographics are summarized in Table 3.

### 3.2. Medical Discipline, Clinical Indication and Causative Pathogen

The majority of referrals for the vOPAT episodes were from surgical wards (*n* = 30, 85.7%), with the orthopedic surgery and trauma surgery wards accounting for most of the discharges (Table 3). Bone and joint infections (*n* = 30, 85.7%) were the most common indication for vOPAT (Table 3). In the majority of the vOPAT episodes, methicillin-resistant *Staphylococcus epidermidis* (*n* = 23, 65.7%) was the isolated causative pathogen (Table 3).

### 3.3. Vancomycin Administration, Target Attainment and Adherence to Dose Adjustment Protocol

Vancomycin was initiated in the hospital as intermittent infusion in most cases (*n* = 34, 97.1%). In one patient (2.9%), vancomycin was started immediately as continuous infusion. The median time and median dose per day to the first therapeutic plasma concentration since start of vancomycin were 74.5 h (range 30.0–230.0) and 2875 mg (range 1250–4000), respectively. For the 11 patients with augmented renal clearance at the start of vancomycin therapy, the median time and median dose per day to the first therapeutic plasma concentration were 74.0 h (range 60.0–230.0) and 3000 mg (range 1680–4000), respectively, as compared to 75.0 h (range 30.0–204.0) and 2000 mg (range 1250–4000) for the patients without augmented renal clearance.

The median time to therapeutic plasma exposure when switching from intermittent to continuous administration of vancomycin was 24.0 h (range 5.0–165.0). A PICC (*n* = 33, 94.3%) was the main type of venous access device used, next to one Midline catheter (2.9%) and one venous port (2.9%).

**Figure 2:**
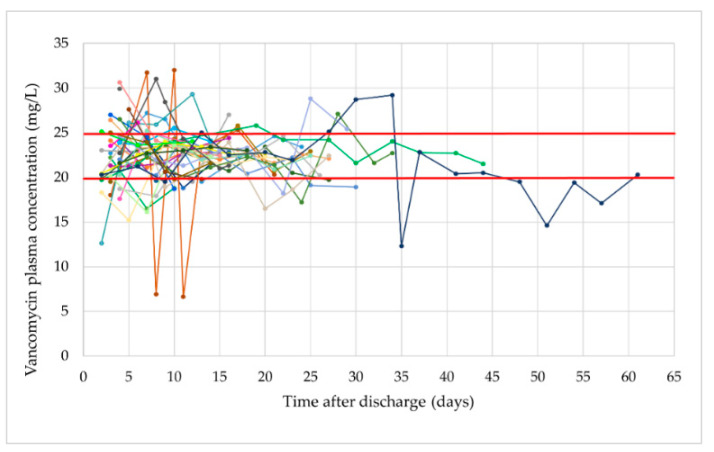
Vancomycin plasma concentrations measured during vOPAT per patient (*n* = 203). Red lines: therapeutic range for vancomycin plasma concentrations when administered via continuous infusion (i.e. 20-25 mg/L).

A total of 206 follow-up consultations at the outpatient clinic were registered. The median number of follow-up consultations per vOPAT episode was five (range 1–19). In total, 203 vancomycin plasma concentrations were measured during these follow-up consultations. Measured plasma concentrations per patient are illustrated in Figure 2. The median vancomycin plasma concentration measured was 22.5 mg/L (range 6.6–32.0). Of all the plasma concentrations, 31.5% (*n* = 64) were non-therapeutic; the number of subtherapeutic (*n* = 34, 16.7%) and supratherapeutic (*n* = 30, 14.8%) plasma concentrations was similar. The median number of non-therapeutic plasma concentration per vOPAT episode was two (range 0–8). Results regarding target attainment are summarized in Table 4.

For 44 (68.8%) of the non-therapeutic concentrations, the vancomycin dose was adjusted. In total, 51 dose adjustments were made; three were considered as urgent and a new elastomeric pump was immediately compounded by the hospital pharmacy.

Patients achieved a therapeutic plasma concentration following a dose adjustment in 31 (60.8%) of the 51 dose adjustments. After two dose adjustments (3.9%), no new plasma concentration was determined because of the end of therapy. Compliance with recommendations from the dose adjustment protocol provided by the OPAT team were 55.6% (*n* = 10) and 45.2% (*n* = 14) in case of patients achieving non-therapeutic and therapeutic plasma concentrations after the dose adjustment, respectively (Table 4).

### 3.4. Treatment Duration, Length of Hospital Stay and Avoided Hospitalization Days

The median length of hospital stay prior to vOPAT was 22 days (range 13–57). The median duration of a vOPAT episode and median total treatment duration with vancomycin were 18 days (range 4–63) and 43 days (range 13–92), respectively. In total, the 35 vOPAT episodes with vancomycin resulted in 746 avoided hospitalization days.

### 3.5. Clinical Outcome, Adverse Events and Hospital Readmissions

One patient (2.9%) experienced a non-vOPAT-related adverse event, i.e., neutropenia. In two vOPAT episodes (5.7%), patients experienced ADEs, including neutropenic fever and a drug reaction with eosinophilia and systemic symptoms (DRESS). These three patients were readmitted during their vOPAT course and vancomycin therapy was stopped. In the remaining 32 vOPAT episodes, all patients were assessed as clinically cured (100%). Two patients (5.7%) suffered from LRAEs (catheter migration (*n* = 1) and phlebitis (*n* = 1)). These patients were temporarily readmitted for insertion of a new catheter, but finished their vOPAT course as planned at home. The patient who suffered from phlebitis initially had a Midline catheter, which was subsequently replaced by a PICC.

### 3.6. Patients’ Satisfaction and Experience

An e-mail address was available in the electronic health record for 12 patients treated between January 2018 and October 2021. The electronic patient survey was therefore sent to these 12 patients and was completed by seven patients, resulting in a response rate of 58%. Patients reported to be very satisfied (*n* = 5, 71.4%) or satisfied (*n* = 2, 28.6%) with their vOPAT course. All patients indicated to be satisfied with the vOPAT service in general and the possibility to continue their treatment at home. One patient reported to be immobile, which made the biweekly hospital visits difficult to organize. One patient reported long waiting times at the outpatient clinic, but also stated that this does not outweigh the advantages of treatment at home.

## 4. Discussion

Long-term therapy with vancomycin is frequently required for indications such as bone and joint infections [12,23,24]. In our study, it was shown that this treatment can be completed at home in an effective and safe way, based on a well-structured vOPAT program providing vancomycin continuous infusion with elastomeric pumps and biweekly follow-up of plasma exposure. Our vOPAT program was successful in attaining clinical cure in 100% of the reviewed vOPAT episodes, with ADEs and LRAEs both occurring in 5.7% of them. In addition to safety, quality of life, such as comfort and mobility, was reported to be satisfying in a limited survey.

Inpatient vancomycin monitoring relies on routine TDM and physician or pharmacist supervision. In contrast, proper monitoring of vancomycin in the outpatient setting still remains quite challenging due to patient immobility, poor access to laboratory facilities, poor transmural communication or geographic isolation [3]. These barriers can lead to default of monitoring, and the risk of lower vancomycin trough levels and inadequate therapy or higher vancomycin trough levels and increased toxicity (i.e., nephrotoxicity). Therefore, we organize and monitor vOPAT within a structured care path including key factors such as strict patient selection, ID consultation, sufficient information provision for all parties involved and biweekly follow-up visits at the outpatient clinic.

Using continuous infusion of vancomycin in our vOPAT program offers some practical advantages over intermittent infusion [25,26]. For example, TDM is more convenient since plasma concentrations are considered as stable, allowing TDM at any given time point. Additionally, only one nursing visit per day is required, as compared to the twice-daily visits needed for intermittent administration [2,25,26,27]. There is no difference in clinical effectiveness between intermittent and continuous infusion of vancomycin, as investigated specifically in the outpatient population by Verall et al. and Shakeraneh et al. [26,27]. Furthermore, there is evidence for vancomycin being less nephrotoxic when administered continuously [6]. Indeed, studies comparing nephrotoxicity of intermittent versus continuous administration, also in the OPAT setting, report continuous infusion being equally or less nephrotoxic [16,25,27]. While incidences of nephrotoxicity during vOPAT described in literature range from 5% to 28.5%, no cases were reported in this study [3,18,25,27,28,29,30,31]. Low prevalence of (highly) supratherapeutic plasma concentrations and the strict biweekly follow-up might contribute to the absence of nephrotoxicity in our study [28].

A higher median daily dose of vancomycin was required to achieve therapeutic vancomycin levels in patients with augmented renal clearance. This should be interpreted with caution given the low number of patients. However, augmented renal clearance indeed has the potential to result in subtherapeutic plasma levels of vancomycin. To prevent therapeutic failure, dose increases are warranted [22,32].

Diamantis et al. reported that vancomycin, together with flucloxacillin, are the two antibiotics the most responsible for adverse events in OPAT [2]. Vancomycin treatment is also described as an independent risk factor for developing ADEs in OPAT [4,18]. Consequently, adequate monitoring for ADEs is indicated during vOPAT. Our vOPAT program combines daily monitoring by the home care nurse, who communicates his/her findings to the OPAT coordinator through a home care follow-up form, with biweekly follow-up consultations at the outpatient clinic to ensure a close monitoring. This strict follow-up might contribute to the low ADE rate observed in our vOPAT program; ADEs occurred in only 5.7% (*n* = 2) of the vOPAT episodes. In contrast, ADE rates of 11–19% have been described in other vOPAT programs [1,3,15,33,34]. Next to ADEs, two patients in this study experienced LRAEs (5.7%), leading to a total adverse event rate of 11.4%. In the study of Voumard et al., where vancomycin was also administered continuously via elastomeric pumps, an adverse event rate (including ADEs and LRAEs) of 16% was reported [1]. Notably, for inpatients with a PICC or a Midline catheter for treatment with vancomycin, LRAE rates up to 17.9% and 19.9%, respectively, have been reported [15]. Although guidelines allow the use of a Midline catheter for vOPAT, it is recommended in the literature, and also preferred in our vOPAT program, to use a central venous route for home administration of vancomycin to avoid complications such as phlebitis [2,5,15]. Despite this preference, one patient was discharged with a Midline catheter, presumably due to inexperience at the start-up of the program. Indeed, this patient subsequently experienced phlebitis, probably caused by the peripheral administration of vancomycin, and required readmission.

The median vancomycin plasma concentration was within the therapeutic range, with a value of 22.5 mg/L (range 6.6–32.0). Of all the measured plasma concentrations, 68.5% was assessed as therapeutic when strictly applying a therapeutic range of 20.0–25.0 mg/L. This assessment might be too strict since small deviations outside the therapeutic range do not necessarily require a dose adjustment. Considering a 5% and 10% deviation, 80.8% and 89.7% of the plasma concentrations, respectively, are within the therapeutic range.

For all vOPAT episodes, patients were assessed as clinically cured with the exception of three vOPAT episodes, where therapy was stopped early because of vOPAT- or non-vOPAT-related complications. Different clinical failure rates were reported in the literature for vOPAT ranging from 3% up to 40% [3,26,27]. Heterogeneity in ambulatory management and follow-up and differences in definitions for assessing clinical failure are possible factors explaining this wide range.

Different approaches have been described in the literature for the management of OPAT in general and more specifically for vOPAT. Grattan et al. found low ADE and treatment failure rates of 11% and 3%, respectively, with the use of a virtual vancomycin monitoring clinic coordinated by nurses. The nurses provided communication and follow-up on vancomycin plasma concentration measurements and the plan of care by phone using an electronic-based monitoring system [3]. Shah et al. proved a beneficial impact of an overseeing clinical pharmacist on adherence to laboratory monitoring recommendations after OPAT discharge for patients without infectious disease supervision. This could possibly lead to fewer adverse events and hospital readmissions [20]. A review by an infectious disease expert prior to discharge with OPAT is suggested by the Infectious Diseases Society of America (IDSA) guidelines on management of OPAT [15]. Infectious disease consultation, as well as a multidisciplinary approach, are recurring components in different OPAT practices and are also the standard of care in the OPAT program of the University Hospitals Leuven [3,13,35,36]. The coordination of care is managed by a clinical pharmacist, who among other things monitors laboratory findings during the biweekly follow-up in close cooperation with the infectious disease specialist. Moreover, all aspects of an OPAT care bundle composed by Muldoon et al., such as outpatient monitoring, program review, patient selection and education, are covered in our vOPAT program [35]. Consequently, the achievement of all these criteria may explain the low ADE, LRAE and treatment failure rates observed in this study, and thus resulting in a safe and effective ambulatory vancomycin treatment.

Strict follow-up with biweekly visits to the hospital can, however, be experienced as a burden for patients, especially for those who are immobile, live far from the hospital or have limited access to transportation to the hospital. This problem was also mentioned by two patients in our limited e-survey. Reducing vancomycin monitoring, and thus hospital visits, from a biweekly to a weekly frequency could be a possible solution in reducing patient burden. Importantly, guidelines suggest at least weekly monitoring of vancomycin exposure and renal function during vOPAT, and this is also the general practice in most vOPAT programs [15,17]. Notably, despite achieving an adequate general target attainment, 35.3% of the patients included in our study still achieved a non-therapeutic plasma concentration after a dose adjustment. Therefore, before considering switching to a weekly monitoring frequency, dose adjustments should be performed more accurately with the aim of better and faster achievement of therapeutic plasma concentrations. This can be realized by refining and increasing adherence to the dose adjustment protocol in the future. Even better would be a population pharmacokinetic model-informed precision dosing-based approach [37]. A more home-based approach for monitoring, comparable with the vOPAT program of Grattan et al., with blood sample collection in collaboration with local laboratories, the general practitioner or the home care nurse, could be another way to relieve the burden of frequent hospital visits [3].

This study has a few limitations. First, the patient survey was sent to only 12 patients due to a lack of registered e-mail addresses in the patient record. Moreover, the survey could only be completed online and not on paper. In the future, patients should be informed of the survey at the start of their vOPAT and given the choice of completing it electronically or via paper. Second, it is unclear whether results of this mono-center study can be generalized since there is no standardization of OPAT practices. In this study, clinical cure was assessed after one month, which might be another limitation considering that recurrent infection can still occur after this period, especially for periprosthetic joint infections [38,39]. Last, the sample size of this study was rather small, which may impact the accuracy of incidence rate estimations.

## 5. Conclusions

vOPAT comes with the challenge of monitoring vancomycin plasma exposure, ADEs and LRAEs in the outpatient setting. The vOPAT program implemented in the University Hospitals Leuven, based on a structured OPAT care path prior to discharge and close ambulatory follow-up after discharge, resulted in successful clinical outcomes with a low rate of adverse events alongside patient satisfaction. Future work should explore reducing the frequency of follow-up consultations at the outpatient clinic to reduce the burden on patients.

## Figures and Tables

**Table 1:** Inclusion and exclusion criteria for the vOPAT program of the University Hospitals Leuven.

**Medical, Infection-Related Criteria**		
	Need for vancomycin antimicrobial therapy	
	Monotherapy with vancomycin	
	Proven infectious focus or diagnosis	
	Directed therapy against an identified micro-organism with susceptibility to vancomycin	
	At least first dose of vancomycin administered in the hospital	
	Treatment with oral antimicrobial agent not possible or appropriate	
		e.g., no oral antimicrobial with same spectrum and sufficient bio-availability
	Infection is biochemically and clinically stable with a predictable course	
		Declining C-reactive protein since start antibiotic treatment
		Afebrile for at least 48 h
**Medical, Non-Infection-Related Criteria**		
	No psychological or cognitive disease or disability	
	No active intravenous drug use	
	No planned hospital admission for (additional) surgery within 7 days after OPAT discharge	
**Patient-Related Criteria**		
	No stay in a nursing or retirement home	
	Self-sufficient patient with adequate cognitive and psychosocial function or sufficient professional support at home	
	Hygienic and adequate home situation	
**Vancomycin-Specific Criteria**		
	Administration by continuous infusion	
	Last two steady-state plasma concentrations within therapeutic range (20–25 mg/L)	
	Central venous line (PICC or venous port)	

PICC: peripherally inserted central catheter; OPAT: outpatient parenteral antimicrobial therapy.

**Table 2:** Dose adjustment protocol for interpretation of vancomycin plasma concentrations during vOPAT.

Plasma Concentration	Recommendations
<15 mg/L	Stop current elastomeric pump. Search for cause of subtherapeutic plasma concentration. Contact the clinical pharmacist for supplementary advice. Administer a new loading dose. Restart continuous infusion with a new elastomeric pump with the correct maintenance dose immediately after administering the loading dose. The new elastomeric pump is compounded by the hospital pharmacy. The patient can be discharged from the outpatient clinic after connecting the new elastomeric pump. From the next day on, the home care nurse changes the elastomeric pumps. After maximum one day, the patient returns to the outpatient clinic for a check-up.
15–17.5 mg/L	Increase dose with 15–25% per 24 h. A new elastomeric pump is compounded by the hospital pharmacy. When receiving the new elastomeric pump, stop the current elastomeric pump. The patient can be discharged from the outpatient clinic after connecting the new elastomeric pump. From the next day on, the home care nurse changes the elastomeric pumps. After maximum two days, the patient returns to the outpatient clinic for a check-up.
17.5–20 mg/L	Increase dose with 5–15% per 24 h. Discharge patient from the outpatient clinic with current elastomeric pump. An elastomeric pump with the new increased dose will be compounded by the home care nurse at the next elastomeric pump exchange. After maximum four days, the patient returns to the outpatient clinic for a check-up.
20–25 mg/L	Maintain the same dose. Discharge patient from the outpatient clinic with current elastomeric pump. After maximum four days, the patient returns to the outpatient clinic for a check-up.
25–27.5 mg/L	Decrease dose with 5–15% per 24 h. Discharge patient from the outpatient clinic with current elastomeric pump. An elastomeric pump with the new decreased dose will be compounded by the home care nurse at the next elastomeric pump exchange. After maximum four days, the patient returns to the outpatient clinic for a check-up.
27.5–30 mg/L	Stop current elastomeric pump. Decrease dose with 15–25% per 24 h. A new elastomeric pump is compounded by the hospital pharmacy The patient can be discharged from the outpatient clinic after connecting the new elastomeric pump. From the next day on, the home care nurse changes the elastomeric pumps. After maximum two days, the patient returns to the outpatient clinic for a check-up.
>30 mg/L	Stop current elastomeric pump. Consider admission depending on clinical condition and renal function.

**Table 3:** Summary of demographic, clinical and treatment-related data of included vOPAT episodes.

**Demographics**		
vOPAT episodes, *n*		35
Patients, *n*		32
	Patients with 2 vOPAT episodes, *n*	3
Male/female, *n*		23/12
Age (years), median (range)		61 (11–75)
eGFR (at start vancomycin therapy), CKD-EPI, mL/min/1.73 m^2^, median (range)		90.0 (45.0–188.0)
**Medical Discipline**		
Surgical, *n* (%)		30 (85.7)
	Orthopedic surgery, *n* (%)	15 (42.9)
	Trauma surgery, *n* (%)	13 (37.1)
	Urology, *n* (%)	1 (2.9)
	Vascular surgery, *n* (%)	1 (2.9)
Internal medicine, *n* (%)		3 (8.6)
	Hematology, *n* (%)	1 (2.9)
	General internal medicine, *n* (%)	1 (2.9)
	Nephrology, *n* (%)	1 (2.9)
Pediatric, *n* (%)		2 (5.7)
**Clinical Indication**		
Bone and joint infections, *n* (%)		30 (85.7)
(Catheter-related) blood stream infection, *n* (%)		3 (8.6)
(Endo)vascular infection, *n* (%)		2 (5.7)
**Causative Pathogen(s)**		
*S. epidermidis*, *n* (%)		21 (60.0)
*S. epidermidis* + *E. faecalis*, *n* (%)		1 (2.9)
*S. epidermidis* + *E. faecium*, *n* (%)		2 (5.7)
*S. epidermidis* + *S. capitis* + *Bacillus*, *n* (%)		1 (2.9)
*S. epidermidis* + *S. capitis*, *n* (%)		1 (2.9)
(Methicillin resistant) *S. aureus*, *n* (%)		3 (8.6)
*S. aureus* + *E. faecalis*, *n* (%)		1 (2.9)
*S. capitis* + *S. salivarius*, *n* (%)		1 (2.9)
*S. hominis* + *S. epidermidis* + *S. warneri*, *n* (%)		1 (2.9)
*S. hominis* + *S. epidermidis* + *S. warneri* + *S. pettenkoferi* + *Arthrobacter* species, *n* (%)		1 (2.9)
*S. hominis*, *n* (%)		1 (2.9)
*E. faecalis* + *E. faecium*, *n* (%)		1 (2.9)

eGFR: estimated glomerular filtration rate; vOPAT: vancomycin in outpatient parenteral antimicrobial therapy.

**Table 4:** Summary of data on therapeutic drug monitoring, clinical outcome and adverse events.

**Target Attainment**		
Total number of vancomycin plasma concentrations measured during vOPAT		203
Total number of therapeutic plasma concentrations during vOPAT (%)		139 (68.5)
Total number of non-therapeutic plasma concentrations during vOPAT (%)		64 (31.5)
	Total number of subtherapeutic plasma concentrations (%)	34 (16.7)
	Total number of supratherapeutic plasma concentrations (%)	30 (14.8)
Total number of therapeutic plasma concentrations + 5% deviation during vOPAT (%)		164 (80.8)
Total number of therapeutic plasma concentrations + 10% deviation during vOPAT (%)		182 (89.7)
Median vancomycin plasma concentration during vOPAT (mg/L) (range)		22.5 (6.6–32.0)
Median number of non-therapeutic plasma concentrations per vOPAT episode (range)		2 (0–8)
**Adherence to Dose Adjustment Protocol**		
Total number of dose adjustments during vOPAT		51
Therapeutic plasma concentration after dose adjustment, *n* (%)		31 (60.8)
	Compliance with dose adjustment protocol, *n* (%)	14 (45.2)
Non-therapeutic plasma concentration after dose adjustment, *n* (%)		18 (35.3)
	Compliance with dose adjustment protocol, *n* (%)	10 (55.6)
Two consecutive subtherapeutic plasma concentrations despite dose increase, *n* (%)		2 (11.1)
Two consecutive supratherapeutic plasma concentrations despite dose reduction, *n* (%)		6 (33.3)
Switch from subtherapeutic to supratherapeutic plasma concentration after dose increase, *n* (%)		6 (33.3)
Switch from supratherapeutic to subtherapeutic plasma concentration after dose reduction, *n* (%)		2 (11.1)
Switch from therapeutic to subtherapeutic plasma concentration after dose reduction, *n* (%)		2 (11.1)
No new plasma concentration following dose adjustment, *n* (%)		2 (3.9)
**Treatment Duration, Length of Hospital Stay and Avoided Hospitalization Days**		
Length of hospital stay (days), median (range)		22 (13–57)
Vancomycin treatment duration before discharge (days), median (range)		20 (9–47)
Duration vOPAT episode (days), median (range)		18 (4–63)
Total vancomycin treatment duration (days), median (range)		43 (13–92)
**Clinical Outcome and vOPAT-Related Adverse Events**		
Clinical cure (*n* = 32) *, *n* (%)		32 (100) *
Readmissions with stop vOPAT, *n* (%)		3 (8.6)
	Readmissions non-vOPAT-related, *n* (%)	1 (2.9)
	Readmissions because of ADEs, *n* (%)	2 (5.7)
Temporarily readmissions with continuation of vOPAT, *n* (%)		3 (8.6)
	Readmission because of difficulties regulating vancomycin plasma concentrations, *n* (%)	1 (2.9)
	Readmission because of LRAEs, *n* (%)	2 (5.7)
LRAEs, *n* (%)		2 (5.7)
	Phlebitis, *n* (%)	1 (2.9)
	Catheter migration, *n* (%)	1 (2.9)

* For 3 patients, the clinical outcome could not be recorded, as vOPAT was stopped early after hospital readmission. ADE: adverse drug event; LRAE: line-related adverse event; vOPAT: vancomycin in OPAT.

## Data Availability

The data underlying this article are available in the article.
